# COL28 promotes proliferation, migration, and EMT of renal tubular epithelial cells

**DOI:** 10.1080/0886022X.2023.2187236

**Published:** 2023-03-08

**Authors:** Linlin Li, Hong Ye, Qiaoling Chen, Lixin Wei

**Affiliations:** Department of nephrology, Fujian Medical University, Union Hospital, Fuzhou, Fujian, China

**Keywords:** Type XXVIII collagen, obstructive nephropathy, epithelial-mesenchymal transition, interstitial fibrosis, type VI collagen

## Abstract

Type XXVIII collagen (COL28) is involved in cancer and lung fibrosis. COL28 polymorphisms and mutations might be involved in kidney fibrosis, but the exact role of COL28 in renal fibrosis is unknown. This study explored the function of COL28 in renal tubular cells by examining the expression of COL28 mRNA and the effects of COL28 overexpression in human tubular cells. COL28 mRNA expression and localization were observed in normal and fibrotic kidney tissues from humans and mice using real-time PCR, western blot, immunofluorescence, and immunohistochemistry. The consequences of COL28 overexpression on cell proliferation, migration, cell polarity, and epithelial-to-mesenchymal transition (EMT) induced by TGF-β1 were examined in human tubular HK-2 cells. COL28 expression was low in human normal renal tissues, mainly observed in the renal tubular epithelial cells and especially in proximal renal tubules. COL28 protein expression in human and mouse obstructive kidney disease was higher than in normal tissues (*p* < 0.05) and more significant in the UUO2-Week than the UUO1-Week group. The overexpression of COL28 promoted HK-2 cell proliferation and enhanced their migration ability (all *p* < 0.05). TGF-β1 (10 ng/ml) induced COL28 mRNA expression in HK-2 cells, decreased E-cadherin and increased α-SMA in the COL28-overexpression group compared with controls (*p* < 0.05). ZO-1 expression decreased while COL6 increased in the COL28-overexpression group compared with controls (*p* < 0.05). In conclusion, COL28 overexpression promotes the migration and proliferation of renal tubular epithelial cells. The EMT could also be involved. COL28 could be a therapeutic target against renal- fibrotic diseases.

## Introduction

The last 25 years saw the growth and aging of the world’s population, leading to major changes in epidemiologic trends, including an increase in the incidence and prevalence of chronic kidney disease (CKD). Globally, from 1990 to 2016, CKD incidence increased by 89% (to reach 21,328,972), CKD prevalence increased by 87% (to reach 275,929,799), and CKD-related mortality increased by 98% (to 1,186,561) [1].

Glomerulosclerosis and interstitial fibrosis inevitably occur during the development of chronic kidney diseases, eventually leading to renal failure. The characteristics of renal fibrosis include the destruction of renal tissue structure, excessive accumulation of extracellular matrix in the renal interstitium, and the loss of renal function [2]. The mechanism of renal fibrosis involves enhanced oxidative stress response, apoptosis of renal intrinsic cells and immune cells, inflammatory response, proliferation, activation of fibroblasts, and transformation of epithelial cells into fibroblasts [3]. A key feature of renal interstitial fibrosis is the epithelial-mesenchymal transition (EMT) and the secretion of extracellular matrix (ECM) components [4].

The collagen family includes several proteins widespread throughout the body and are important for tissue and organ scaffolding, cell adhesion and migration, cancer, angiogenesis, tissue morphogenesis, and repair [5]. Different types of collagens are found in the ECM and play vital roles in the structural characteristics of different tissues, constitute scaffolds for cell adhesion and migration, and play important roles in various diseases [6,7]. Many collagen-related diseases are caused by gene mutations encoding a collagen polypeptide chain [8–12]. These mutations can lead to a spectrum of conditions since different collagen proteins also directly regulate the phenotypic state of cells by transmitting signals to mesenchymal cells, epithelial cells, and endothelial cells that affect their proliferation, differentiation, polarization, and survival [13–15].

Type XXVIII collagen (COL28) is a recently described (in 2006) homotrimeric molecule. Its ɑ-chain contains a 528-amino acid collagenous domain and two von Willebrand factor A (VWA) modules involved in protein-protein interactions [16]. The expression of COL28 displays a heterogeneous localization around Schwann cells and nerve bundles, and COL28 is a marker of non-myelinated zones of the peripheral somatosensory system [17]. Zhao et al. [18] reported that mutations in the COL28A1 gene were associated with spontaneous preterm birth. Chen et al. [19] showed that COL28A1, as a biomarker, is associated with benefits from immune checkpoint inhibitor treatment in patients with melanoma. Yang et al. [20] screened 13 key genes related to the prognosis of glioblastoma multiforme, among which COL28A1 was the most important. COL28 is involved in lung fibrosis and might be a therapeutic target [21]. Besides its role in various diseases, COL28 is expressed in the kidney [16], and COL28A1 polymorphisms appear to modulate the development of diabetic nephropathy [22], a disease involving renal fibrosis.

The authors’ team has found a missense mutation in COL28 that might cause tubulointerstitial fibrosis and uremia. A proband (33-years old) of a uremic family underwent sequencing, revealing a missense mutation in COL28 that resulted in a change in amino acid 722. Bioinformatics predicted that this change would directly affect the function of COL28. A renal biopsy revealed chronic tubulointerstitial disease (data not shown). Therefore, it was hypothesized that COL28 could play an important role in maintaining the normal function of renal tubules. Therefore, this study explored the functions of COL28 in tubular cells by examining the expression of COL28 mRNA and localization of its protein in kidney tissue in humans and mice with normal kidneys and fibrotic renal diseases. The COL28 gene was overexpressed in human tubular HK-2 cells to determine the consequences of COL28 overexpression on cell proliferation, migration, cell polarity, and EMT induced by TGF-β1. The results could provide a better understanding of fibrotic kidney disease and provide novel potential therapeutic targets.

## Materials and methods

### Animals

C57BL/6J SPF male mice aged 8 weeks and weighing 25–30 g were obtained from the Shanghai SLAC Laboratory Animal Co. (Shanghai, China). The animals were housed in three mice per cage and with free access to chow and water. All animals were kept inadequate sanitary conditions. The animals were divided into three experimental groups (six mice/group): the control group, which was submitted only to anesthesia (isoflurane 2% inhalation) and laparotomy; the unilateral ureteral occlusion (UUO) 1-week group, which was submitted to anesthesia (isoflurane 2% inhalation) and left UUO [23], then sacrificed (by cervical dislocation after inhalation anesthesia with isoflurane 2%) on the 7^th^ day after surgery; the UUO 2-week group, which was submitted to anesthesia (inhalation anesthesia with isoflurane 2%) and left UUO [23], then sacrificed (by cervical dislocation after inhalation anesthesia with isoflurane 2%) on the 14^th^ day after surgery. All animal experiments were approved by the Animal Ethics Committee of Fu Jian Medical University (approval #2020070101) and performed in compliance with the Guidelines for the Care and Use of Laboratory Animals published by the US National Institutes of Health (NIH Publication, 8th Edition, 2011).

### Tissue collection

Eight patients who underwent nephrectomy due to renal cancer in the urology department of Fujian Medical University Union Hospital were enrolled from January 2018 to December 2019. Normal non-cancerous tissues were taken 3 cm away from cancer. Renal tissues were stained by hematoxylin and eosin (H&E) to exclude structural abnormalities and cancer. All patients were diagnosed with renal tumors. All underwent total nephrectomy.

Eight patients who underwent surgical treatment for obstructive nephropathy were also enrolled during the same period. Paraffin-embedded tissue sections from these patients were used for Mason staining and immunohistochemistry. Their diagnosis was nephrolithiasis and severe hydronephrosis.

The hospital’s ethics committee approved the study (2020ky070). All methods were carried out in accordance with relevant regulations and guidelines. All participants signed the informed consent form.

### Histopathological examination

Harvested tissues were fixed in 10% neutral buffered formaldehyde solution for 24 h and then paraffin-embedded before sectioning at 3 µm. After deparaffinization using xylene, the sections were stained with H&E and Masson trichrome. Five non-overlapping fields were selected at 200× magnification (E600; Nikon, Tokyo, Japan) and examined by two pathologists separately. The total renal histopathological score (0, normal; 1, mild impairment; 2, moderate impairment; 3, severe damage) was calculated based on eight items: interstitial fibrosis, interstitial edema, interstitial infiltration, tubular atrophy, red tube, protein casts, tubule vacuolar degeneration, and tubular dilatation [24]. The blue-stained (i.e., fibrotic) areas were quantified by morphometric analysis using Image-Pro Plus 6.0 software (Media Cybernetics, Silver Spring, MD, USA).

### Immunohistochemistry

Immunohistochemistry was performed on 3-μm formalin-fixed paraffin-embedded (FFPE) sections to detect the protein expression and localization of COL28 in human kidneys. The primary antibody used in this study was rabbit anti-collagen XXVIII (1:1000, ab188533, Abcam, Cambridge, The United Kingdom). The sections were incubated overnight in primary antibody buffer at 4 °C. After washing with PBS, they were stained with Elivision^TM^ super HRP (Mouse/Rabbit) IHC Kit (Maixin Biotech, Ltd., Fuzhou, China). After immunostaining, the sections were counterstained with hematoxylin. Representative pictures were captured using microscopy (Leica Microsystems, Wetzlar, Germany).

### Immunofluorescence

Immunofluorescence was performed on 4-μm FFPE sections to detect the co-localization of COL28 with AQP1, AQP3, and THP1 in normal human kidneys. The sections were treated in a repair box with EDTA antigen repair buffer (pH 8.0), and antigen repair was carried out using a microwave-based antigen retrieval technique. Sections were observed under a fluorescence microscope, and images were collected.

### Cell culture

HK-2 cells (human kidney proximal tubular cells line) were from the American Type Culture Collection (Manassas, VA, USA) and cultured in Dulbecco’s modified Eagle’s medium/F12 medium (Hyclone, Logan, UT, USA) containing 10% FBS (Hyclone) and 1% antibiotics (100 U/ml penicillin and 100 μg/ml streptomycin) (Life Technologies Co., Grand Island, NY, USA). The cells were incubated at 37 °C in a humidified incubator with 5% CO_2_. For TGF-β1 treatment, the cells were cultured in a serum-free medium with or without recombinant human TGF-β1 (CA#10021, PeproTech, Rocky Hill, NJ, USA) with different concentrations.

### COL28 overexpression

The COL28A1-expressing lentivirus vector (Lv-COL28A1) and the negative universal control (Lv-NC) containing the green fluorescent protein EGFP and a puromycin resistance gene were constructed by Shanghai Genechem Co., Ltd. (Shanghai, China). Before infection with the virus, the HK-2 cells were seeded onto a 6-well plate (1.0 × 105 cells/well) and allowed to grow to 30% confluency. The Lv-COL28A1 and Lv-NC were transfected into the cells with an enhanced infection solution (Shanghai Genechem Co., Ltd.) and polybrene (Sigma, St. Louis, MO, USA). The transfected HK-2 cells were selected with puromycin (3 μg/ml), and the expression of the fluorescence protein EGFP was used to monitor the infection efficiency by fluorescence microscopy after 72 h. The Lv-COL28A1-positive cells were designated as COL28-OE, and the Lv-NC cells were designated as COL28-NC.

### Cell viability assay

HK-2 cells were seeded into 96-well culture plates (1 × 10^4^ cells/well) and treated with various concentrations of TGF-β1 (0,1, 2, 5, 10, and 15 ng/ml) for 24 h. Cell viability was evaluated using a Cell Counting Kit-8 (CCK-8; Genview, Australian). Absorbance was measured at 450 nm (Spectra Max i3X, Molecular Devices, LLC, Sunnyvale, CA, USA).

### Colony formation assay

The cells were divided into three groups: the control group (CON; HK-2 cells), COL28-NC, and COL28-OE. First, the cells in the three groups were made into single-cell suspensions using 0.25% trypsin. Afterward, the cells were coated onto plates (1.5 × 103 cells per well) and incubated at 37 °C and 5% CO2 for 2 weeks. After 2 weeks, the cells were washed two times with PBS, fixed with 5 mL of methanol (Sigma, St. Louis, MO, USA), and stained with 0.1% Giemsa solution (Sigma) within 10 min.

### Wound healing assay

The mobility of the cells was evaluated by a wound-healing assay. The cells in the three groups (CON, COL28-NC, and COL28-OE) were incubated in 6-well tissue culture plates for 24 h to form a monolayer. A 20-µL pipette tip was used to scratch a line and remove the cells. Each well was washed twice with 1 mL of PBS to remove the detached cells. Then, 2 mL of DMEM/F12 was added to each well, and the plate was incubated for 24, 48, and 72 h. Cells were washed twice with PBS. The width of the cell-free space was measured at 0, 24, 48, and 72 h using a microscope (Carl Zeiss GmbH, Oberkochen, Germany).

### Transwell assays

Migration assays were carried out using Transwell chambers (8 µm pore size, Corning Inc., Corning, NY, USA). The cells in the three groups (CON, COL28-NC, and COL28-OE) suspended in a serum-free medium were loaded onto the upper chamber, while a medium containing 10% FBS was added to the lower chamber. After 48 h of incubation at 37 °C, the Transwell chambers were removed from the incubator. The cells in the upper chamber were removed, and the migrated cells in the lower chamber were stained with crystal violet for 20 min. Finally, the number of migrated cells was counted under a microscope.

### Quantitative real-time PCR

Total RNA was isolated using Trizol (Invitrogen Inc., Carlsbad, CA, USA). RNA (2 µg) was reverse-transcribed using the high-capacity cDNA Reverse Transcriptase kit (Roche Applied Science, Penzberg, Germany). The primers are listed in Table 1. Real-time PCR amplification was performed using the SYBR Green PCR Master Mix Kit (Invitrogen). The relative quantity of mRNA was normalized to β-actin and calculated using the 2^-ΔΔCt^ method.

**Table 1.** Primers for quantitative real-time PCR primers.

Genes	Sequences, 5’->3’	Length of the product (bp)
*COL28A1*	F: CAGCCCTTCAGTTTAGCAG	173
	R: ATCCTTACGCCCTTCTCTC	
*CDH1*	F: AGTCACTGACACCAACGATAAT	205
	R: ATCGTTCACTGGATTTGTG	
*ACTA2*	F: CCTGAAGTACCCGATAGAACATG	273
	R: TCTCCAGAGTCCAGCACGAT	
*TJP1*	F: AAAGAGAAAGGTGAAACACTGC	135
	R: TTTTAGAGCAAAAGACCAACCG	
*ACTB*	F: GGGCCGGACTCGTCATAC	144
	R: CCTGGCACCCAGCACAAT	

*COL28A1*: collagen XXVIII gene; *CDH1*: E-cadherin gene; *ACTA2*: α-smooth muscle actin gene; *TJP1*: zonula occludens-1 gene; *ACTB*: β-actin gene.

### Western blot

Western blot was used to detect COL28 protein expression and localization in the normal renal cortex and medulla according to the manufacturer’s protocol. The densitometric analysis of the images was performed using Image J software.

### Statistical analysis

Data were shown as means ± standard deviations and analyzed using GraphPad Prism 6.0 (GraphPad Software Inc., San Diego, CA, USA). The unpaired two-tailed *t*-test was used for comparison between the two groups. One-way analysis of variance (ANOVA) and Tukey’s *posthoc* test was used to compare more than two groups. Two-sided *p*-values <0.05 were considered statistically significant.

## Results

### COL28 is mainly expressed and located in proximal renal tubules of normal kidney tissue

**Figure 1.**
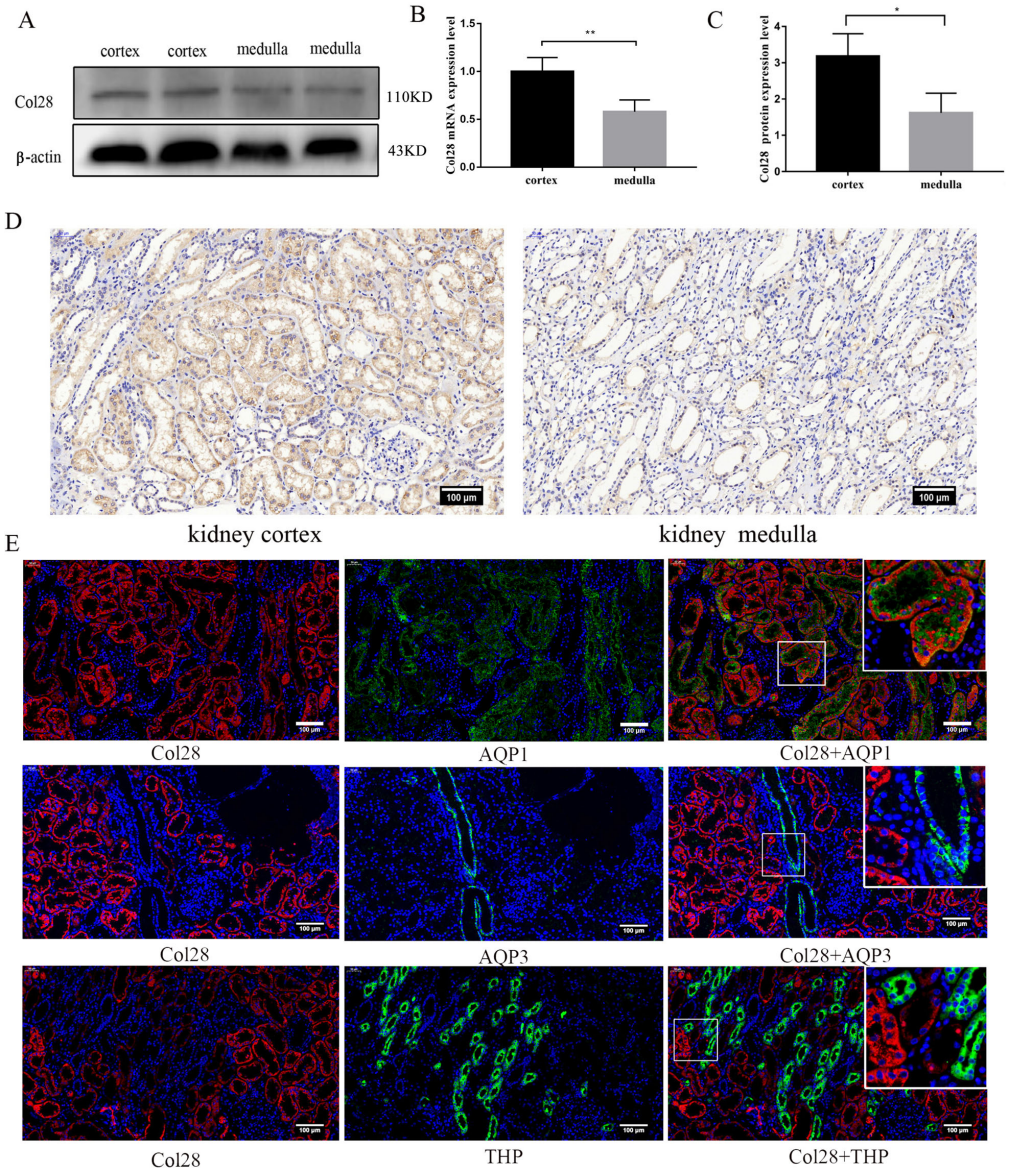
Expression and localization of COL28 in human normal kidney tissues. COL28 expression in the cortex and medulla of normal kidney tissue was analyzed by western blot (A) and immunohistochemistry (D). COL28 mRNA (B) and protein (C) expression were quantified and calibrated with the expression of β-actin. *p < 0.05, **p < 0.01. The localization of COL28 in normal kidney tissues (E). The experiments were repeated three times using eight samples each time. Data are presented as mean ± SD. Bar = 100 µm. Red represents COL28, green represents AQP1, AQP3, and THP, and blue represents the cell nuclei.

Western blot and qPCR were used to detect the COL28 mRNA and protein expression and localization in the normal renal cortex and medulla. The results showed that COL28 mRNA and protein expression in the renal cortex was higher than in the renal medulla (Figure 1(A–C)), suggesting that COL28 was mainly expressed in the renal cortex. Immunohistochemistry showed that the COL28 protein was mainly expressed in renal tubules, especially in the cortex, and there was little staining in medullary tubules (Figure 1(D)). Glomeruli and renal interstitium were rarely stained. Immunofluorescence showed that COL28 co-located with AQP1, the marker protein of the proximal tubule, but not with THP, the marker protein of the Loop of Henle, and AQP3 of the collector, suggesting that COL28 was mainly expressed in the proximal tubule of kidney in normal human tissues (Figure 1(E)).

### COL28 expression is increased in human and mouse renal tissue with obstructive kidney disease

**Figure 2.**
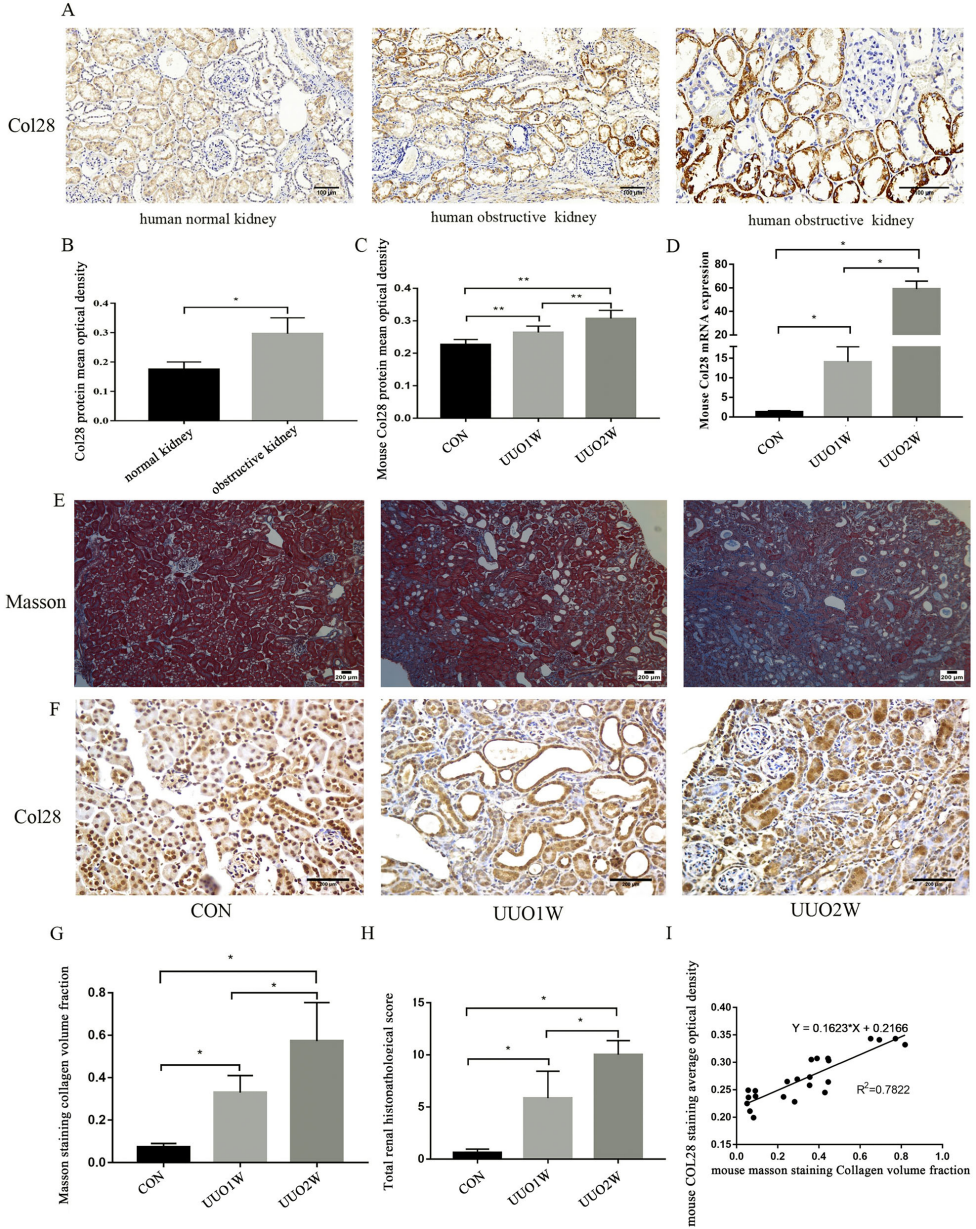
COL28 expression in human obstructive kidney tissue and mouse obstructive kidney tissue. Image of normal and obstructive renal tissue stained by COL28 immunohistochemistry (A). Bar = 100 µm. Quantification of the expression levels of COL28 proteins in normal and obstructive kidney tissues (B). Graphical representation of the protein (C) and mRNA (D) expression levels of COL28 in mouse control, UUO 1-week, and UUO 2-week groups. *p < 0.05, **p < 0.01. Image of mason (E) and COL28 immunohistochemistry staining (F) in the mouse control, UUO 1-week, and UUO 2-week groups. Bar = 200 µm. Total renal histopathological score (G) and Masson straining surface area (H) in the mouse control, UUO 1-week, and UUO 2-week groups. (I) Correlation between the total renal histopathological score and Masson straining surface area. *p < 0.05.

COL28 was evaluated in obstructive nephropathy. COL28 staining in renal tubules increased with the aggravation of obstructive lesions (Figure 2(A–B)). In the UUO mouse models, H&E and Masson’s staining showed that renal tubules were dilated, flattened, and detached in the UUO1W group. A few inflammatory cells infiltrated the stroma. The expression of COL28 was significantly increased in the UUO1W group, and all tubules were stained, especially the dilated tubules (Figure 2(C–F)). In the UUO2W group, renal tubule atrophy and necrosis were obvious. Renal interstitial fibrosis was obvious. The staining of COL28 was most obvious in the renal tissues of the UUO2W group, especially in the atrophic tubules, showing block-like staining (Figure 2(C–F)). Compared with the CON and UUO1W groups, the total renal histopathological score and fibrotic area percentage of the UUO2W group were higher (both *p* < 0.05), while the expression of COL28 in the UUO2W group was increased compared with the UUO1W group (Figure 2(G–H)). We collected the pathological sections of eight mice in the control, UUO1W, and UUO2W groups, carried out COL28 immunohistochemistry and Masson staining, and analyzed the correlation between the optical density of COL28 staining and the collagen volume fraction of Masson staining. The results showed that the optical density of COL28 staining in mice was positively correlated with the volume fraction of collagen. It is suggested that with the aggravation of renal fibrosis in mice, COL28 staining is also gradually increased (Figure 2(I)).

### Overexpression of COL28 promotes HK-2 cell proliferation

**Figure 3.**
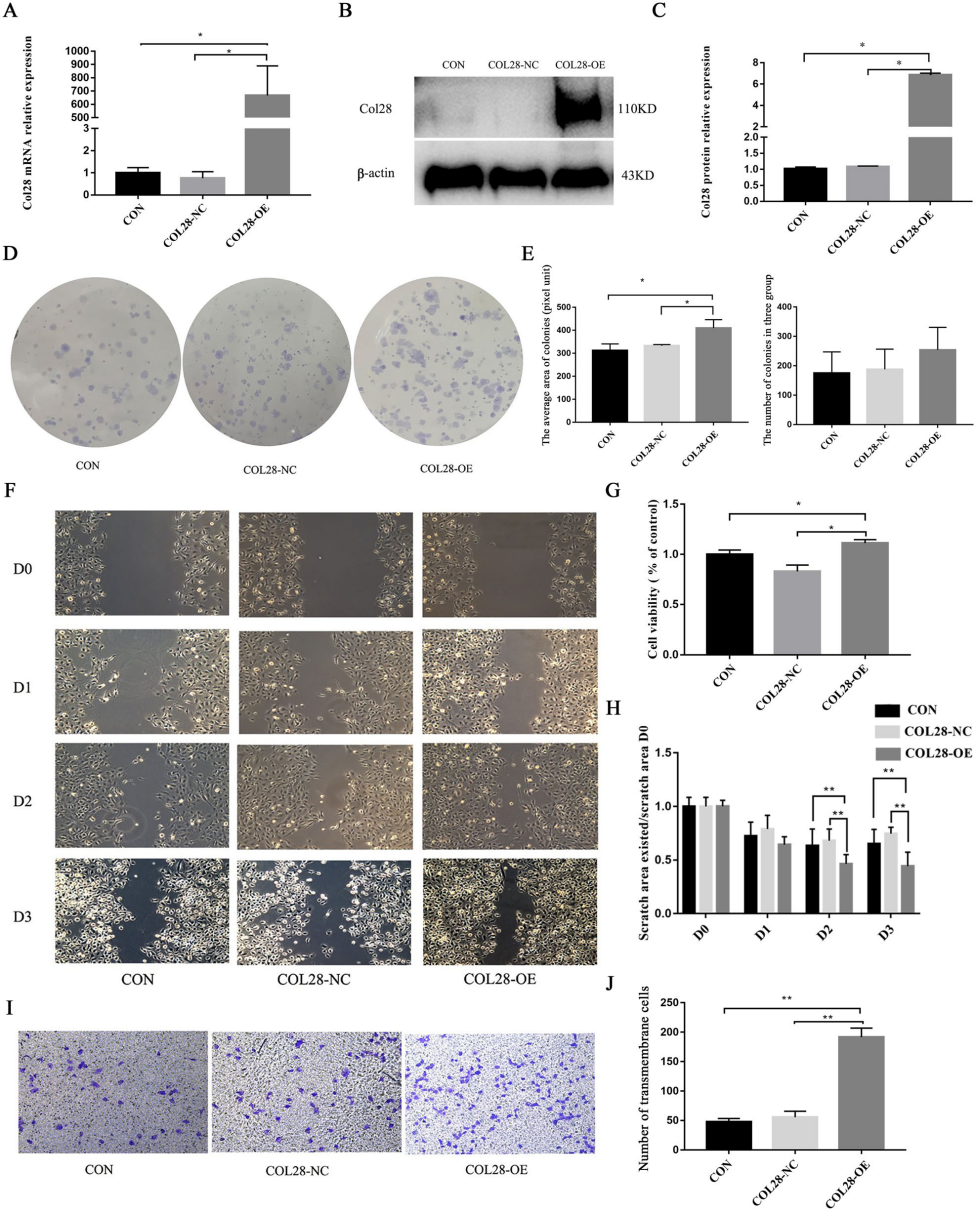
Overexpression of COL28 promotes HK-2 cell proliferation and migration. The mRNA (A) and protein (B-C) expression of COL28 in HK-2 cells. Effect of COL28 overexpression on the proliferation of HK-2 cells detected by the plate cloning method (D-E). *p < 0.05. Comparison of the cell viability in the CON, COL28-NC, and COL28-OE groups by CCK-8 (G). *p < 0.05. Cell migration ability in the three groups by the wound healing test (F&H) **p < 0.01. Cell migration ability in the three groups by the Transwell assay (I-J). **p < 0.01. All experiments were performed three times.

To evaluate the effect of COL28 overexpression on HK-2 proliferation, COL28 was overexpressed in HK-2 cells (Figure 3(A–C)). The effect of COL28 overexpression on the proliferation of HK-2 cells was detected by the plate cloning method. There were no differences in the numbers of colonies among the COL28-OE, CON, and COL28-NC groups, but the diameter of each colony was larger in the COL28-OE group compared with the two other groups (*p* < 0.05) (Figure 3(D–E)). The cell viability of the COL28-OE group was higher than in the CON and COL28-NC groups (*p* < 0.05) (Figure 3(G)). Therefore, COL28 overexpression can promote the proliferation of HK-2 cells.

### Overexpression of COL28 promotes HK-2 cell migration

The cell wounding assay was used to detect the effect of overexpression of COL28 on the migration ability of HK-2 cells. Compared with the CON and COL28-NC groups, the scratch areas of the cells in the COL28-OE group were reduced, and the cell migration ability was the strongest (Figure 3(F,H)). The Transwell assay showed that the number of cells passing through the membrane in the COL28-OE group was higher than in the CON and COL28-NC groups (*p* < 0.01) (Figure 3(I–J)). These results suggest that COL28 overexpression can promote the migration ability of HK-2 cells.

### Overexpression of COL28 promotes HK-2 cell EMT induced by TGF-β1

The preliminary experiments showed that the cell viability was decreased with TGF-β1 15 ng/ml compared with the controls (*p* < 0.05) (Figure 5(A)). The cells gradually changed from the original oval and paving-stone shape to a fibroblast-like appearance with the increase in TGF-β1 concentration (Figure 5(B)). E-cadherin mRNA expression decreased with the increasing TGF-β1 concentration, while the mRNA expression of α-SMA and COL28 increased (Figure 5(C)).

**Figure 4.**
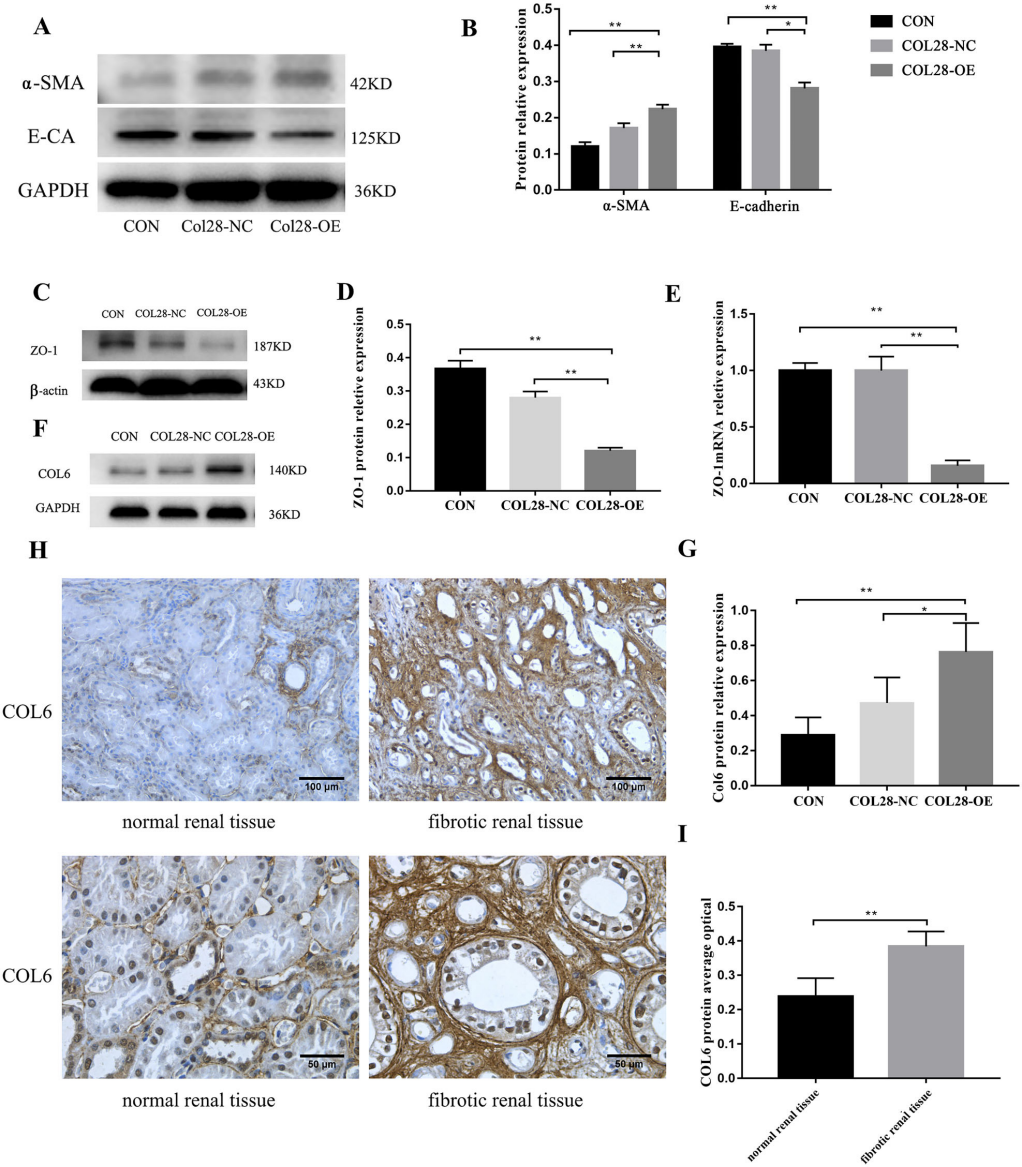
Effect of overexpression of COL28 on the expression of E-cadherin and α-SMA protein in HK-2 cells after induction with 10 ng/ml TGF-β1 (A-B). *p < 0.05 **p < 0.01. Overexpression of COL28 in HK-2 cells inhibited ZO-1 protein (C-D) and mRNA (E) expression. **p < 0.01. COL6 protein expression levels in the CON, COL28-NC, and COL28-OE groups (F-G). *p < 0.05 **p < 0.01. COL6 protein expression in human fibrotic renal tissue was higher than in human normal renal tissue (H, I). **p < 0.01. All experiments were performed three times.

After 10 ng/ml TGF-β1 induction, α-SMA expression in the COL28-OE group was higher than in the CON and COL28-NC groups (*p* < 0.01). The expression of E-cadherin in the COL28-OE group was lower than in the CON and COL28-NC groups (*p* < 0.01) (Figure 4(A–B)). These results suggest that the expression of COL28 aggravates the EMT induced by TGF-β1. The relative expression levels of ZO-1 mRNA and protein in the COL28-OE group were lower than in the COL28-NC group (*p* < 0.01) (Figure 4(C–E)). The overexpression of COL28 could significantly inhibit the expression of ZO-1 in HK-2 cells.

**Figure 5.**
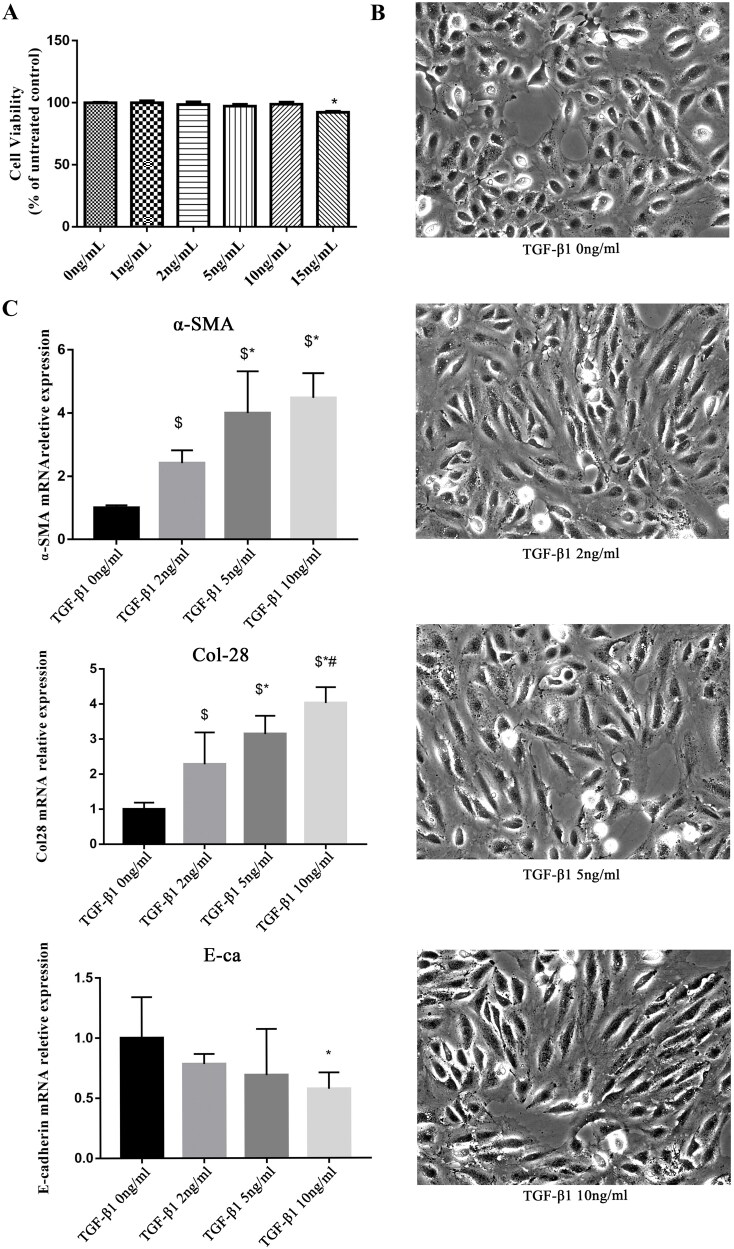
COL28 Overexpression promotes HK-2 cell EMT induced by TGF-β1. HK-2 cell viability was detected by CCK-8 under stimulation of TGF-β1 at different concentrations (A). Morphological changes in HK-2 cells are induced by different concentrations of TGF-β1 (B). Expression of E-cadherin, α-SMA, and endogenous COL28 mRNA in HK-2 cells induced by TGF-β1 at different concentrations (C). $*p* < 0.01 vs. TGF-β1 0 ng/ml, *p < 0.01 vs. TGF-β1 2 ng/ml, #*p* < 0.01 vs. TGF-β1 5 ng/ml. All experiments were performed three times.

### COL28 overexpression increases COL6 protein expression in HK-2 cells

After the stimulation of HK-2 cells with TGF-β1 10 ng/ml, the expression levels of the COL6 protein in the COL28-OE group were higher than in the CON and COL28-NC groups (Figure 4 (F–G)). Only a small amount of COL6 was expressed in the normal human renal interstitium, while in the human fibrotic kidney, COL6 staining in the stroma was significantly enhanced (*p* < 0.01) (Figure 4(H–I)). The results suggest that the overexpression of COL28 could promote the expression of COL6 in HK-2 cells.

## Discussion

COL28 is involved in cancer and lung fibrosis [19–21]. Polymorphisms and mutations in COL28 might be involved in kidney fibrosis [22], but the role of COL28 in renal fibrosis is unknown. Therefore, this study aimed to explore the function of COL28 in tubular cells by examining the expression of COL28 mRNA and localization of its protein in kidney tissue and the effects of COL28 overexpression in human tubular cells. The results strongly suggest that COL28 expression is high in renal fibrosis, both in human and mouse models. COL28 overexpression promotes the proliferation, migration, and EMT of renal tubular epithelial cells and increases COL6 expression. Therefore, COL28 might be a therapeutic target against renal fibrotic diseases.

COL28 is the latest discovered collagen and thus has been only sparsely investigated. In the collagen superfamily, COL28 is similar to COL6 in the structure of its α-chain. They belong to the class of beaded filament-forming collagen. COL6 plays several key functions in various tissues, including promoting tumor growth and progression and regulating autophagy and cell differentiation [25–27], but whether COL28, which has a structure similar to COL6, also has similar functions is unknown. Reese-Petersen et al. [28] found that pro-COL28 levels in peripheral blood were high in patients with lung cancer and patients with heart failure with preserved ejection fraction. In addition, Pro-COL28 levels are high in diseases featuring a high ECM turnover [28]. COL28 could be involved in fibroproliferative conditions of the heart and lungs [28]. In the present study, immunohistochemistry showed that COL28 was mainly located in the proximal tubules of the renal cortex. Immunofluorescence showed that COL28 co-located with AQP1 (expressed on proximal tubules) but not with THP and AQP3 (expressed in the Loops of Henle and collecting ducts, respectively). These results confirmed that COL28 is expressed in the kidney, especially in the proximal tubules of the kidney in normal kidney tissues. The mechanism of renal fibrosis involves enhanced oxidative stress response, apoptosis of renal intrinsic cells and immune cells, inflammatory response, the proliferation of renal cells, activation of fibroblasts, and transformation of epithelial cells into fibroblasts [3]. A key feature of renal interstitial fibrosis is the epithelial-mesenchymal transition (EMT) and the secretion of extracellular matrix (ECM) components [4]. Cell cycle dysregulation leading to excessive renal cell proliferation is a feature of fibrotic kidney diseases and is a potential treatment target against renal fibrosis [29]. The present study suggests that COL28 can activate cell proliferation in kidney diseases. Although it is not the only factor affecting cell proliferation, COL28 is involved in the process. Future studies should investigate the mechanisms leading to increased COL28 expression and its involvement in the proliferation of renal tubular epithelial cells.

The induction of UUO in mice is a standard model of nonimmunological tubulointerstitial fibrosis [30]. The main renal damage caused by UUO is tubular damage and interstitial fibrosis. In the pathological tissues of human obstructive kidney disease collected in the present study, the COL28 staining on the proximal renal tubules was significantly enhanced compared with controls. The mouse UUO model confirmed that with the aggravation of interstitial fibrosis, the COL28 mRNA and protein expression levels in renal tissue were increased. These results are consistent. Therefore, the expression of COL28 is high in renal interstitial fibrosis, and its expression is correlated, to some extent, with the severity of the obstruction. Nevertheless, COL28 expression was reduced in renal tissues with severe fibrosis, i.e., in which the renal proximal tubules are completely detached, necrotic, lost, infiltrated by inflammatory cells, or completely replaced by fibrotic tissue. Therefore, it can be speculated that COL28 shows a process of first increased expression and finally decreased expression during renal tubular injury and interstitial fibrosis. Future studies will have to examine that hypothesis.

During urinary tract obstruction, resident fibroblasts become activated, and myofibroblasts are produced from several sources, including epithelial cells (*via* the epithelial-mesenchymal transition [EMT]), pericytes, endothelial cells, and bone-marrow-derived cells [31–34]. Myofibroblasts observed in the kidney after UUO can originate from tubular cells after EMT, but also pericytes and resident fibroblasts. Activated fibroblasts and myofibroblasts cells play important roles in interstitial fibrosis [31]. In the present study, COL28 overexpression in HK-2 cells promoted the proliferation of HK-2 cells and increased the migration ability of renal tubular epithelial cells. These results indicated that overexpressing COL28 could activate the renal tubular epithelial cells and promote the generation of myofibroblasts from renal tubular epithelial cells.

EMT is an important pathologic pathway involved in renal interstitial fibrosis [35]. The first and most critical step in EMT is reducing E-cadherin expression. Once E-cadherin is reduced, cell-to-cell adhesion decreases, the cells lose their tight intercellular junctions, and finally lose epithelial cell polarity; after EMT, the cells also show increased α-SMA expression, suggesting that the cells acquired a myofibroblast-like phenotype, enhancing their proliferation and invasive potentials [35,36]. At last, basement membrane degradation is also a feature of kidney EMT [37]. ZO-1 is one of the important proteins belonging to tight junctions (TJs) [38,39]. ZO-1 is important to maintain the polarity of epithelial cells and is also involved in forming the cytoskeleton, cell proliferation, and differentiation [40,41]. During EMT, the decrease of ZO-1 is accompanied by a decrease of Claudin, occludin expression, and the cleavage of E-cadherin on the plasma membrane [42–44]. COL28 overexpression could inhibit the expression of ZO-1 and E-cadherin in HK-2 cells, and promote the expression of α-SMA, suggesting that COL28 can affect the integrity of tight junctions of epithelial cells, destroy the adhesion between cells, inhibit the formation of renal tubular epithelial cells polarity, and promote EMT [43], thus aggravating kidney injury.

Accumulation of the extracellular matrix (ECM) protein significantly contributes to glomerulosclerosis and TIF. ECM is mainly synthesized and secreted by fibroblasts/myofibroblasts in the glomerulus and tubulointerstitium. Growing evidence suggests that EMT of renal tubular epithelial cells is one source of matrix-producing fibroblasts and myofibroblasts, which is one of the potential mechanisms involved in kidney fibrosis [45]. Clinical and *in vitro* studies suggest that COL6 might stimulate cell proliferation, leading to tissue fibrosis [46]. Deposition of COL6 was high in patients with kidney diseases compared with control patients, and COL6 was higher in diabetic glomeruli, associated with α-actin-positive myofibroblasts [47–49]. In fibrotic renal tissue, the expression of COL6 was significantly upregulated. Under the induction of TGF-β1, COL28 overexpression could further stimulate the production of the COL6 protein in HK-2 cells, suggesting that COL28 can aggravate renal interstitial fibrosis by inducing the expression of COL6.

This study has some shortcomings. First, only the functional changes of HK-2 cells after overexpression of COL28 were studied, but knockdown was not studied. The expression of COL28 in HK-2 cells is already very low under physiological conditions. In the qRT-PCR experiment, the average CT value was greater than 32. If COL28 is further knocked down at such a low expression level, it would be difficult to evaluate the effect after knockdown, and it would be difficult to carry out subsequent cell function experiments. Secondly, COL28 could aggravate renal interstitial fibrosis by promoting cell proliferation, migration, and EMT, but the exact mechanisms were not explored. A recent transcriptomics study by the authors suggests that COL28 overexpression aggravates renal interstitial fibrosis by increasing the EMT of renal tubular epithelial cells; interfering with HKDC1 expression appears to reverse the COL28-induced EMT and alleviate fibrosis [50]. Transcriptomic analyses of the changes of HK-2 cells overexpressing COL28 are still necessary under different pathological conditions, and overexpression/silencing of different genes to explore the meaningful differentially expressed genes and cell pathways related to proliferation, migration, and EMT.

In conclusion, COL28 expression is high in renal fibrosis, both in human and mouse models. COL28 overexpression promotes the proliferation and migration of renal tubular epithelial cells and increases COL6 expression. The EMT could also be involved in the process. Therefore, COL28 might be a therapeutic target against renal fibrotic diseases.

## Data Availability

All data generated or analyzed during this study are included in this published article.
